# Estimates of Insulin Out-of-Pocket Cap–Associated Prescription Satisfaction, Adherence, and Affordability Among Medicare Beneficiaries

**DOI:** 10.1001/jamanetworkopen.2022.51208

**Published:** 2023-01-13

**Authors:** Minghui Li, Jing Yuan, Kevin Lu

**Affiliations:** 1 Department of Clinical Pharmacy and Translational Science, University of Tennessee Health Science Center, Memphis; 2 Department of Clinical Pharmacy, School of Pharmacy, Fudan University, Shanghai, China; 3 Department of Clinical Pharmacy and Outcomes Sciences, University of South Carolina, Columbia

## Abstract

This survey study examines the association of the Inflation Reduction Act of 2022 insulin out-of-pocket cap with prescription satisfaction, adherence, and affordability among Medicare beneficiaries and associated disparities.

## Introduction

Insulin expenditures under Medicare have almost doubled in the last decade, leading to reported cost-related underuse.^1,2^ These reports highlight the issues of insulin affordability and adherence and the urgent need to address them.^1^ The Inflation Reduction Act of 2022 (IRA) proposes to cap insulin out-of-pocket costs at $35 per month.^3^ However, little is known about the potential benefits and equity implications of this policy. We examined the association of the insulin out-of-pocket cap with prescription satisfaction, adherence, and affordability among Medicare-insured insulin users and to identify associated disparities.

## Methods

The University of Tennessee Health Science Center Institutional Review Board approved this survey study. Informed consent was waived because we used deidentified data (for January 1, 2006, to December 31, 2019) from the Medicare Current Beneficiary Survey (MCBS). The MCBS links survey reports to Medicare claims and provides comprehensive information on expenditures and payment sources for prescription drugs. Additionally, the MCBS collects patient-reported data on prescription satisfaction, adherence, and affordability (eMethods in Supplement 1).^4^ This study followed the AAPOR reporting guideline.

Logistic regression analyses were conducted to estimate the association of meeting the $35 insulin out-of-pocket cap with prescription satisfaction, adherence, and affordability by adjusting for patient age, sex, self-reported race and ethnicity, education, marital status, residence, US Census region, general health, low-income subsidy, plan type, diabetes type, and comorbidities. Two-sided *P* < .05 indicated significance. To account for multiple comparisons, Bonferroni corrections were used for subgroup analyses on age, sex, and race and ethnicity. Survey sampling weights were applied for national estimates. SAS version 9.4 (SAS Institute Inc) was used for statistical analysis. Statistical analysis was performed on October 3, 2022.

## Results

Of the 3.1 million insulin users in this survey study covered under Medicare Part D, 1.4 million (45.1%) had insulin out-of-pocket costs of $35 or more per month in 2019 (Figure). The weighted proportion of insulin users who met the $35 insulin out-of-pocket cap increased from 21.7% in 2006 to 44.6% in 2013, and remained stable through 2019. Notable differences in meeting the eligibility criteria for the insulin out-of-pocket cap were identified in terms of age, sex, and race and ethnicity. Medicare beneficiaries who were aged 64 years or younger, women, or racial and ethnic minority individuals were less likely to be eligible for the $35 insulin out-of-pocket cap.

**Figure.**
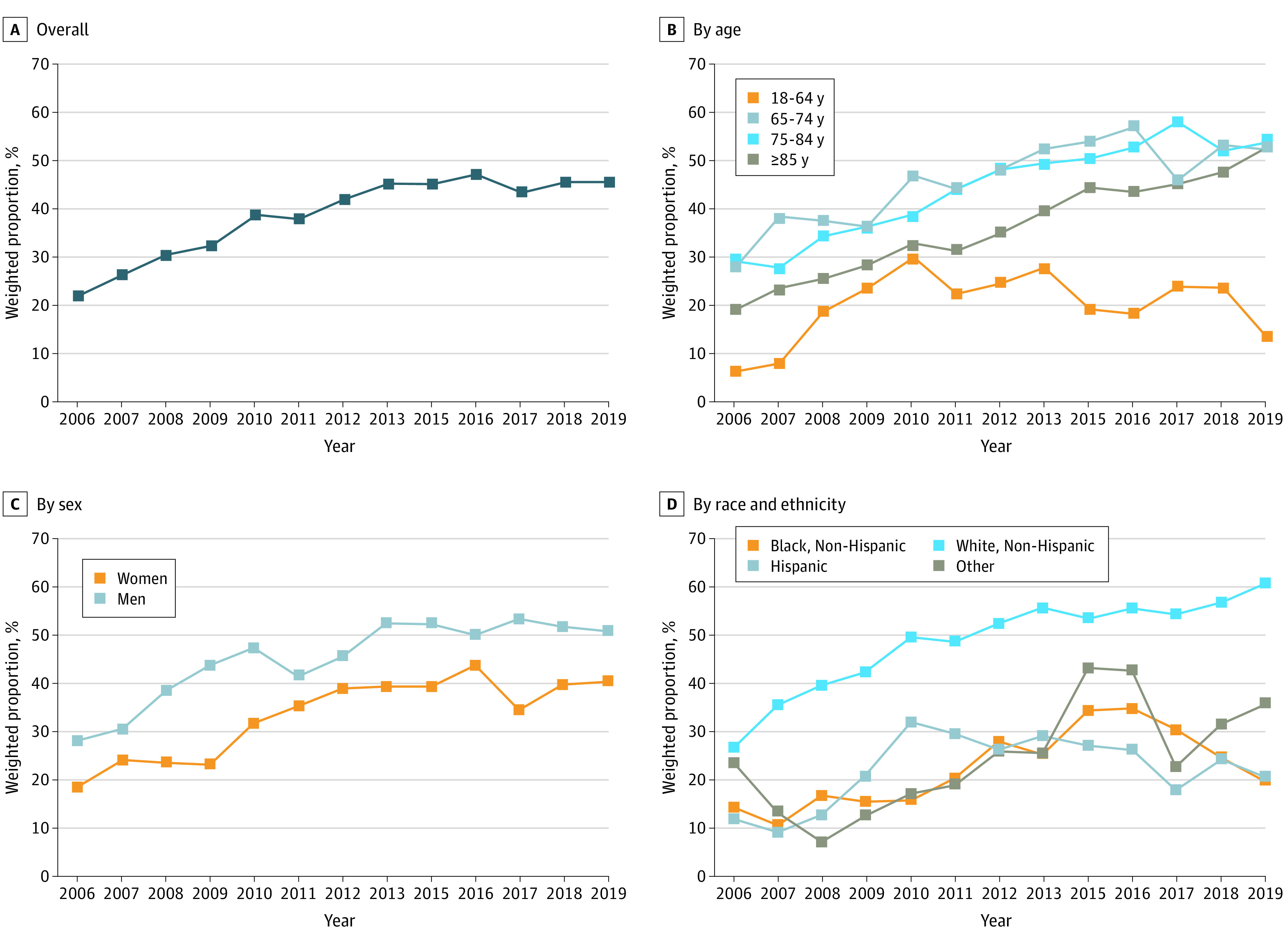
Trends in Weighted Proportions of Insulin Users With Medicare Part D Coverage Who Met the Insulin Out-of-Pocket Cap, 2006 to 2019 A, Overall weighted proportions of insulin users for the study period. B to D, Subgroup analysis by patient age (B), sex (C), and race and ethnicity (D).

Having insulin out-of-pocket costs of $35 or more per month was associated with an 83.2% lower likelihood of prescription satisfaction (odds ratio [OR], 0.17 [95% CI, 0.13-0.22]), a 61.3% lower likelihood of prescription adherence (OR, 0.39 [95% CI, 0.30-0.50]), and a 49.7% lower likelihood of prescription affordability (OR, 0.50 [95% CI, 0.38-0.67]) (Table). High cost-sharing was a substantial barrier to prescription satisfaction, adherence, and affordability in most subgroups.

**Table.** Insulin Out-of-Pocket Cap and Associated Prescription Satisfaction, Adherence, and Affordability for Insulin Users With Medicare Part D Coverage^a^

Subgroup characteristic	Odds ratio (95% CI)		
	Satisfaction	Adherence	Affordability
Overall	0.17 (0.13-0.22)	0.39 (0.30-0.50)	0.50 (0.38-0.67)
Age, y^b^			
18-64	0.14 (0.06-0.32)	0.29 (0.15-0.56)	0.54 (0.26-1.12)
65-74	0.15 (0.09-0.26)	0.39 (0.25-0.61)	0.45 (0.25-0.81)
75-84	0.21 (0.14-0.33)	0.43 (0.24-0.76)	0.52 (0.24-1.14)
≥85	0.13 (0.04-0.42)	0.93 (0.22-3.87)	3.79 (1.18-12.13)
Sex^b^			
Women	0.16 (0.11-0.24)	0.46 (0.30-0.70)	0.55 (0.37-0.83)
Men	0.17 (0.11-0.26)	0.32 (0.20-0.52)	0.46 (0.27-0.79)
Race and ethnicity^b^			
Black, non-Hispanic	0.17 (0.10-0.29)	0.32 (0.16-0.64)	0.33 (0.16-0.70)
Hispanic	0.11 (0.05-0.23)	0.30 (0.14-0.67)	0.39 (0.17-0.85)
White, non-Hispanic	0.16 (0.10-0.24)	0.38 (0.26-0.56)	0.56 (0.37-0.85)
Other^c^	0.22 (0.11-0.43)	0.53 (0.24-1.18)	0.66 (0.26-1.73)

^a^ Multivariable logistic regression models estimated the association of the insulin out-of-pocket cap with each outcome by adjusting for age, sex, self-reported race and ethnicity, education, marital status, residence, US Census region, general health, low-income subsidy, plan type, diabetes type, and comorbidities.

^b^ Bonferroni-corrected CIs to handle multiple comparisons.

^c^ Includes American Indian or Alaska Native, Asian, Native Hawaiian or other Pacific Islander, and more than 1 race or ethnicity.

## Discussion

In this survey study, we observed that the economic burden of insulin increasingly shifted to patients between 2006 and 2019 in the form of high cost-sharing, which is a substantial barrier to insulin access. Our findings suggest that by capping insulin out-of-pocket costs at $35 per month, the IRA will likely benefit nearly half of Medicare-insured insulin users and may improve prescription satisfaction, adherence, and affordability among patients.

This study warrants more equitable eligibility criteria for the IRA insulin cap in the future. Our findings suggest that some populations such as racial and ethnic minority individuals and women are less likely to be eligible for the insulin cap, which may perpetuate inequities in benefitting from this cap in the future. A limitation is that this study may be subject to proxy response bias when participants are not available or unable to respond.

Medicare beneficiaries who reach the IRA insulin cap will have no financial incentives to use lower-priced insulin (eg, synthetic human insulin and biosimilar insulin). This lack of incentive will likely lead to higher total insulin expenditures, from a societal perspective. Policy makers may consider strategies (eg, encouraging use of biosimilar insulin) to control future insulin costs.^5^ Attention should also be paid to expensive medications other than insulin, because patients with diabetes are increasingly being treated with these drugs.

## Notes

Supplement 1.
eMethods

Supplement 2.
Data Sharing Statement
