# Supplementary material for: Estimates of Insulin Out-of-Pocket Cap–Associated Prescription Satisfaction, Adherence, and Affordability Among Medicare Beneficiaries
Source: JAMA Netw Open. 2023 Jan 13;6(1):e2251208. doi: 10.1001/jamanetworkopen.2022.51208 (PMC9857611; doi:10.1001/jamanetworkopen.2022.51208)
Supplement: Supplement 1. — eMethods [file jamanetwopen-e2251208-s001.pdf]

Supplementary Online Content

Li M, Yuan J, Lu K. Estimates of insulin out-of-pocket cap–associated prescription satisfaction, adherence, and affordability among Medicare beneficiaries. *JAMA Netw Open*. 2023;6(1):e2251208. doi:10.1001/jamanetworkopen.2022.51208

eMethods.

This supplementary material has been provided by the authors to give readers additional information about their work.

© 2023 Li M et al. *JAMA Network Open.*

eMethods.

If participants reported being “satisfied/very satisfied” with the amount paid for prescribed drugs, they will be considered satisfied with prescription drugs. Cost-related medication nonadherence is a summary measure that includes: 1) not filling a prescription because it cost too much, 2) delaying getting a prescription filled because the drug cost too much, 3) skipping doses to make the drug last longer, and 4) taking smaller doses than prescribed of a drug to make the drug last longer. If participants reported “never” to all of the above questions, they will be considered adherent to prescription drugs (not have cost-related medication nonadherence). If participants reported “never” spending less money on food, heat, or other basic needs to have money for medicine, they will be considered being able to afford prescription drugs.

© 2023 Li M et al. *JAMA Network Open.*
