# Supplementary material for: Estimates of Insulin Out-of-Pocket Cap–Associated Prescription Satisfaction, Adherence, and Affordability Among Medicare Beneficiaries
Source: JAMA Netw Open. 2023 Jan 13;6(1):e2251208. doi: 10.1001/jamanetworkopen.2022.51208 (PMC9857611; doi:10.1001/jamanetworkopen.2022.51208)
Supplement: Supplement 2. — Data Sharing Statement [file jamanetwopen-e2251208-s002.pdf]

Data Sharing Statement

Li. Estimates of Insulin Out-of-Pocket Cap-Associated Prescription Satisfaction, Adherence, and Affordability Among Medicare Beneficiaries. *JAMA Netw Open*. Published January 13, 2023. doi:10.1001/jamanetworkopen.2022.51208

Data
Data available: No

Additional Information
Explanation for why data not available: The data have been shared by the Centers for Medicare & Medicaid Services (CMS) at https://www.cms.gov/research-statistics-data-and-systems/research/mcbs
